# A Sediment Diagenesis Model of Seasonal Nitrate and Ammonium Flux Spatial Variation Contributing to Eutrophication at Taihu, China

**DOI:** 10.3390/ijerph17114158

**Published:** 2020-06-11

**Authors:** Linda Sarpong, Yiping Li, Eyram Norgbey, Amechi S. Nwankwegu, Yue Cheng, Salifu Nasiru, Isaac Kwesi Nooni, Victor Edem Setordjie

**Affiliations:** 1 Key Laboratory of Integrated Regulation and Resource Development on Shallow Lakes, Ministry of Education, Hohai University, Nanjing 210098, China; lynsarp27@yahoo.com (L.S.); eyramnorgbey@outlook.com (E.N.); asnwankwegu@hhu.edu.cn (A.S.N.); yuec244@gmail.com (Y.C.); 2 College of Environment, Hohai University, Nanjing 210098, China; 3 College of Mechanics and Materials, Hohai University, Nanjing 210098, China; snasiru@hhu.edu.cn; 4 School of Geographical Sciences, Nanjing University of Information Science & Technology, Nanjing 210044, China; nooni25593@alumni.itc.nl; 5 Binjiang College, Nanjing University of Information Science & Technology, No.333 Xishan Road, Wuxi 214105, China; 6 College of Coastal, Harbor and Offshore Engineering, Hohai University, Nanjing 210098, China; eddyseth@yahoo.com

**Keywords:** diagenesis, nitrate, ammonium, sediment flux, water quality, algae, sustainability

## Abstract

Algal blooms have thrived on the third-largest shallow lake in China, Taihu over the past decade. Due to the recycling of nutrients such as nitrate and ammonium, this problem has been difficult to eradicate. Sediment flux, a product of diagenesis, explains the recycling of nutrients. The objective was to simulate the seasonal spatial variations of nitrate and ammonium flux. In this paper, sediment diagenesis modeling was applied to Taihu with Environmental Fluid Dynamics Code (EFDC). Latin hypercube sampling was used to create an input file from twelve (12) nitrogen related parameters of sediment diagenesis and incorporated into the EFDC. The results were analyzed under four seasons: summer, autumn, winter, and spring. The concentration of NH_4_–N in the sediment–water column increased from 2.744903 to 22.38613 (g/m^3^). In summer, there was an accumulation of ammonium in the water column. In autumn and winter, the sediment was progressively oxidized. In spring, low-oxygen conditions intensify denitrification. This allows algal blooms to continue to thrive, creating a threat to water quality sustainability. The sediment diagenesis model, coupled with water quality measured data, showed an average relative error for Total Nitrogen (TN) of 38.137%, making the model suitable. Future studies should simulate phosphate flux and measure sediment fluxes on the lake.

## 1. Introduction

Algal blooms have been the most current problem in the third-largest freshwater lake in China, Taihu, over the past decade. The presence of nitrate (NO_3_) and ammonium (NH_4_) makes algae production thrive [1]. Sediment resuspension has been carefully explored in recent times on the lakes; however, one of the major sources providing nutrients to facilitate resuspension, diagenesis, is still a research gap [2,3,4,5,6,7].

The sediment diagenesis process transforms particulate organic matter (POM) deposited into dissolved matters at the benthic region, which results in the production of diagenesis fluxes. Sediment flux recycles the products of diagenesis and consistently renders the water quality of the lake low [2,8,9]. These diffused nutrients from the sediment bed contribute immensely to eutrophication problems, even after external sources have been substantially reduced [10].

Sediment flux is a major contributor to nutrient recycling in a lake’s ecosystem and in its management. Trends in sediment fluxes have been investigated since before the 1990s, but did not include South America, Africa, and Oceania; however, in 2019, an update on this research was done that considered these areas where the focus was on rivers rather than on lakes [11]. This points out that regardless of extreme sensitivity to seasonal and environmental changes, with lakes, the rate at which sediment flux could contribute immensely to the water quality has still not been explored for better lake management remedies [12,13].

Diagenetic reactions at the sediment bed create a substantial nutrient source. Harmful algal blooms (HAB) are induced by the presence of the two most common nutrients: nitrogen (nitrates, ammonia, and urea) and phosphate. Issues of HABs have been a challenge in many large lakes worldwide over the past decade such as Lake Winnipeg in Canada [14]; Lake Erie in the Laurentian Great Lakes [15]; and Lake Taihu [16], Dianchi [17], and Lake Chaohu [18] in China.

A remedial action of reducing nitrogen and phosphorus loadings through improved wastewater treatment and control of point and non-point source nutrients offer a short term solution. All of the physical, chemical, and biological management approaches traditionally adopted in mitigating the global impact of harmful cyanobacterial blooms (cyanoHABs) analyzed concluded that no remedial step provided a sustainable solution to HABs on a global scale [19,20]. However, these approaches provide more preventive measures in the interim, rather than a long-term sustainable approach [21].

Blooms contain organisms such as algae, which render the lake low in oxygen and also produce toxins that contaminate the water. As a result, aquatic lives are endangered, and the natural cycle that maintains a clean aquatic environment is threatened. After cleaning the lake, a diagenetic reaction can trigger a eutrophic lake that used to be clean to suddenly become green with algae [7,10]. Therefore, sediment diagenesis becomes a crucial aspect of water quality modeling, if long term sustainable solutions are being considered [22,23,24].

**Figure 1:**
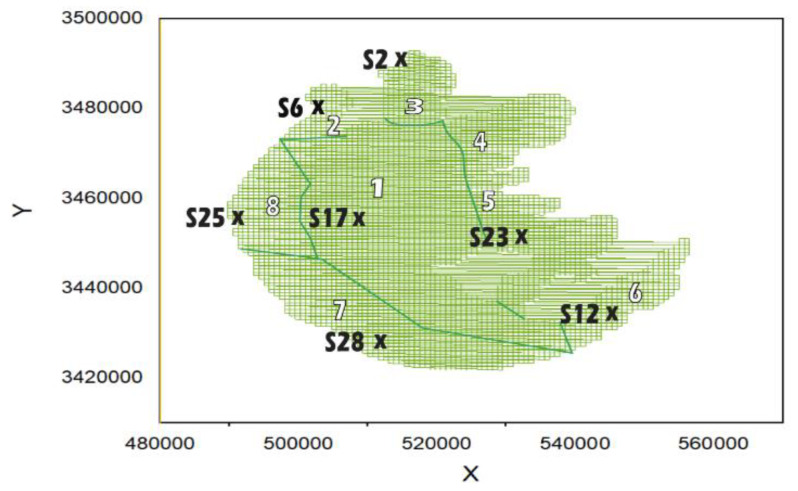
Eutrophic Lake Taihu in its grid form. Zones: (1) Central Zone, (2) Zhushan Bay, (3) Meiliang Bay, (4) Gonghu Bay, (5) East Epigeal Zone, (6) DongTaihu Bay, (7) Southwest Zone, (8) Northwest Zone, and data recording stations (S).

In this paper, sediment diagenesis modeling was applied to the third-largest freshwater lake in China, Lake Taihu (Figure 1), due to its eutrophication issues over the years to understand why the problem has been persistent over a decade, regardless of the countless models that have been produced on it [25]. Large shallow lakes such as Taihu, with its long past of receiving nutrient-rich inflows, are considered to also maintain high rates of internal and external recycling [26,27]. The objective of this research is to model the seasonal spatial variations of nitrate (NO_3_) and ammonium (NH_4_) flux. This contributes to the prevailing algae blooms consistently causing eutrophication on Taihu [23,28,29,30].

## 2. Materials and Methods

### 2.1. The Research Area

Lake Taihu, with its complex shoreline geometries, is connected to 172 rivers or channels and is the third-largest shallow freshwater lake in China [19]. It is located between 119°08′–122°55′ E and 30°05′–32°08′ N, having a catchment area of 36,500 km^2^ with a surface area of 2338 km^2^ [3]. The mean (maximum) depth of the lake is 1.9 m (2.6 m), corresponding to an elevation of 3.0 m [3]. The lake bottom features a flat terrain with a mean geographic gradient of 0°0′19.66″.

A shallow-water area with an average depth of < 1.5 m is sited in the eastern part and accounts for 19.3% of the total surface area. The deepest areas (>2.5 m), occupy nearly 8.4% of the total lake area, and are in the north and west. According to field observations, thermal stratification is not explicitly evident, even though it exists on the lake. Its duration is typically less than one day, as it is easily mixed by wind force [3,31]. Moreover, the temperature difference from the surface to the bottom mostly ranges from 0–1 °C and other cases of 1–4 °C per field monitoring data available from December 2007 to November [32]. The leading wind direction on the lake is southeast in the summer and northwest in the winter at an average wind speed of 3.5–5 m/s [3]. For easy monitoring and management of Lake Taihu, it has been divided into eight subareas: Zhushan Bay, Meiliang Bay, Gonghu Bay, Northwest Zone, Southwest Zone, Central Zone, East Epigeal Zone, and Dongtaihu Bay (Figure 1).

### 2.2. The Role of the Environmental Fluid Dynamics Code

A three-dimensional hydrodynamic model originally developed by John Hamrick called the Environmental Fluid Dynamic Code (EFDC) was utilized to simulate the seasonal variation of sediment flux on Lake Taihu [33]. The model is one of the most widely applied advanced modeling frameworks for simulating hydrodynamics, water quality, eutrophication, and dynamic changes and interactions in sediment transportation in lakes, rivers, and estuaries [18].

It has 22 state variables in the water column and is coupled with 27 state variable sediment diagenesis models. The sediment diagenesis model is based on a recording of the model developed by DiToro and Fitzpatrick [9]. The sediment diagenesis model receives particulate organic matter (POM), in this case, specifically particulate organic nitrogen (PON), which has been deposited from the water column.

EFDC then simulates the diagenesis, and the resulting fluxes of nitrate (NO_3_) and ammonium (NH_4_) escape back into the water column. The water quality model is coupled with sediment diagenesis. This coupling of the models not only enhances predictively, but gives room to simulate long term variations [24]. Countless studies have used this tool, and its demonstration shows its convenience, faster calculation times, accuracy, robustness, and reproducibility [34]. Nevertheless, this approach has not been applied to Lake Taihu.

### 2.3. Principles of Diagenesis Fluxes

The principle of mass conservation governs sediment diagenesis models like water quality models. The diagenesis expression used, which is the kinetic equation for particulate organic nitrogen (PON) is based on the mass balance equation:

Net PON change = − decay of PON − burial + depositional flux(1)

The depositional fluxes settle directly at the lower part of the lake called the lower layer or L2 in EFDC. Diagenesis occurs only in the lower layer. The upper layer, L1(aerobic) is considered as not significant because of its small thickness: L1; $H_{1\;}$= 0.1 cm, while L2; $H_{2\;}$= 10 cm.

(2) $$H_{2\;}\frac{dG_{PON{,}_{i\;}}}{dt}=-k_{PON{,}_{i\;}}\theta_{PON{,}_{i\;}}^{\left(T-20\right)}\;G_{PON{,}_{i\;}}H_{2\;}-\omega_{\;}\;G_{PON{,}_{i\;}}+J_{PON{,}_{i\;}\;}$$

where $G_{PON{,}_{i\;}}$ is the PON concentration in ith G class (Layer 2(g/m^3^) in the anaerobic layer; $k_{PON{,}_{i\;}}$ is the decay rate of the ith G class PON at 20 °C in Layer 2(day^−1^); $\theta_{PON{,}_{i\;}}$ is the constant temperature adjustment for $k_{PON{,}_{i}}$; T is the sediment temperature (°C); and ω is the burial rate (m/day). $J_{PON{,}_{i\;}}$ is the depositional fluxes that serve as a source term to drive the diagenesis process [32].

### 2.4. Constructing the Sediment Diagenesis of Nitrate (NO_3_), Ammonium–Nitrogen Flux Model

Twelve (12) parameters were selected for the sediment diagenesis model chosen through a detailed investigation of the literature, creating an input file with the Latin hypercube sampling method (LHS) (Table 1). The LHS approach generates a sequence for every possible combination of the independent input parameters. It works by taking the range of each independent parameter, dividing the range by the selected realizations, rearranging the values into a random distribution, and then combining the distributions for each independent parameter [35].

A set of 200 parameter combinations was produced by the LHS random sampling and incorporated into the EFDC water quality model by [1,35]. The EFDC nitrate (NO_3_) and NH_4_ flux Taihu model was set up in a horizontal Cartesian coordinate system divided into 4,465,750 m x 750 m square meshes with a uniform grid, as shown in Figure 1. The grid is vertically divided into three layers using *σ* coordinates. The slope of the model lake bottom was less than 0.33, preventing the pressure caused by the *σ* coordinates.

The force gradient error serves as input as the boundary condition of the model by the atmosphere, wind speed and direction, lake flow, water quality, and sediment. The atmospheric conditions and wind farms were based on the Nanjing Geography and Lake Research Institute of the Chinese Academy of Sciences, and data from the National Field Observation and Research Station of the Taihu Lake Ecosystem.

The initial simulation date was 20 June, and assumed as initial. The water level was horizontal and set to the average of the first day of the simulation period. The initial flow rate was set to 0 m/s. Model calculation time was 365 days, and the time step was one (1) minute. The model was run 200 times to ensure standard calibration, accuracy, and reproducibility.

### 2.5. Data Recording Stations

Seven (7) S data recording stations from the National Field Observation and Research Station on Taihu Lake across all eight (8) zones of Figure 1 were used for calibration, namely; (S2) Small Wanli, (S6) Zhu Lake, (S12) East Taihu Lake, (S17) Platform Mountains, (S23) Xu Lake, (S25) V. East, and (S28) Xiaomei Mouth. Since EFDC sediment diagenesis model allows coupling with the water quality model, the available measured water quality values of total nitrogen (TN) for the seven (7) S stations were used. TN was considered because the EFDC sediment diagenesis model uses nitrogen related parameters.

## 3. Results

### 3.1. Seasonal Variation of Nitrate (NO_3_) Flux Analysis

The outcome of the simulation presented 17,856 simulated data points each day for 365 days because of the number of nodes. Due to the large datasets, some days were selected based on the critical times present in each month of the year. The eleventh day was chosen, which was the last day in June, based on the initial time of the simulation because the variation of the fluxes would have started showing after 10 days of waiting.

The 41st day was almost at the end of July, and the 70th day was at the end of August. These selected days would have had ample time to manifest the variations of the fluxes. The same understanding was applied to the 90th, 120th, and 140th days present in September, October, and November, respectively. In addition, the 160th, 185th, and 200th days were calculated from December, January, and February, respectively.

Finally, the 230th, 250th, and the 300th days were also selected based on the initial date of simulation from March, April, and May, respectively. A direct comparison was made at a seasonal timescale. Winter was defined as December–January–February (DJF), spring as March–April–May (MAM), summer as June–July–August, (JJA), and autumn as September–October–November, (SON).

**Figure 2:**
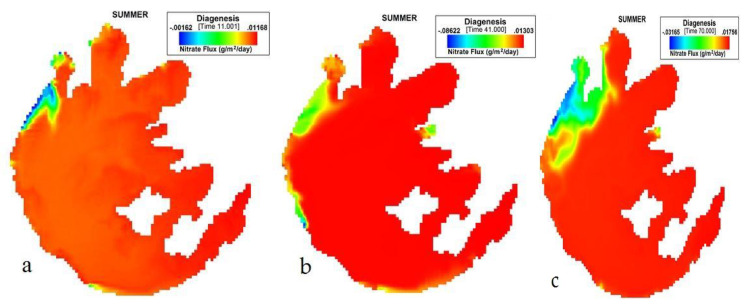
(**a**) 11th, (**b**) 41st, (**c**) 70th day simulation of NO_3_ flux variation during summer at Taihu.

Figure 2 presents the simulation of NO_3_ flux variation during JJA on Taihu. Nitrate flux variation during summer began from the northwest zone of Taihu and a little at the boundary of Zhushan Bay with the lowest influx of −0.06622, an average of 0.011333 (g/m^2^/day), Table 2. The highest flux value during JJA was 0.01756 as shown on the 70th day. There was a gradual rise in the sediment flux of nitrate. Based on the values during this period, the rate of nitrate flux escaping into the water column was very low. 

**Figure 3:**
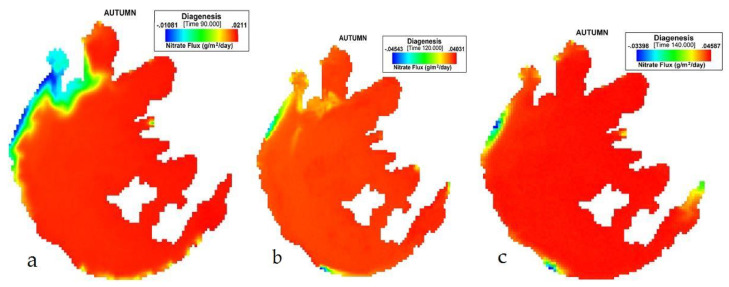
(**a**) 90th, (**b**) 120th, (**c**) 140th day simulation of NO_3_ flux variation during autumn at Taihu.

Figure 3 presents the simulation of NO_3_ flux variation during SON in Taihu. Its sediment flux ranges from 0.018149 to 0.0433 (g/m^2^/day). The lowest influx was −0.04543 (g/m^2^/day), an average of 0.031883 (g/m^2^/day).

**Figure 4:**
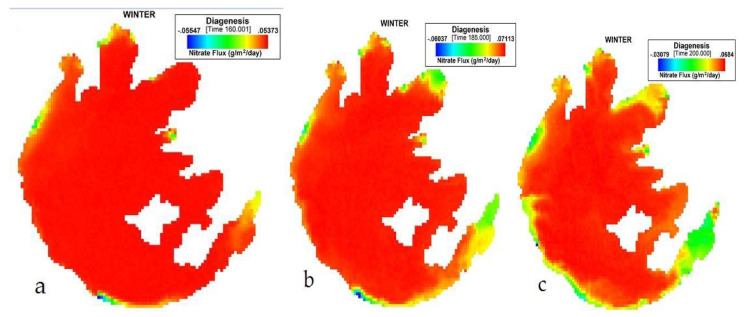
(**a**) 160th, (**b**) 185th, (**c**) 200th day simulation of NO_3_ flux variation during winter at Taihu.

Figure 4 presents the simulation of NO_3_ flux variation during winter on Taihu. During DJF, the nitrate (NO_3_) flux variation extended gradually from all seven boundaries toward the central zone with a range of −0.03079 to 0.0684 (g/m^2^/day), but with the highest average value of 0.0637 (g/m^2^/day). The lowest influx was −0.06037 (g/m^2^/day) with an average of 0.058067 (g/m^2^/day).

**Figure 5:**
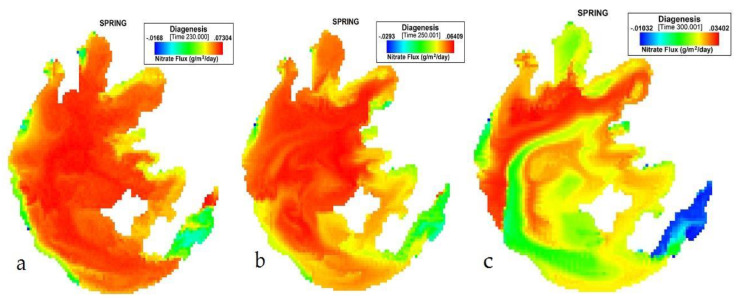
(**a**) 230th, (**b**) 250th, (**c**) 300th day simulation of NO_3_ flux variation during spring at Taihu.

Finally, in spring (MAM), from Figure 5, the lowest influx was −0.0163, with an average of 0.044933. The nitrate flux values started declining from the 231st day to the last day. The average sediment flux values dropped from 0.0618 to 0.0223 (g/m^2^/day).

### 3.2. Seasonal Variation of Concentration Ammonium–Nitrogen (Conc. NH_4_–N) Analysis

The results showed Layer 1 and 2 (L1 and L2, respectively). Due to the negligible nature of L1, the results shown in Table 2 are from L2m which is where diagenesis occurs. Diagenesis of the concentration of NH_4_–N in the sediment–water column increased from 2.744903 to 22.38613 (g/m^3^) (see Table 2). The highest efflux was experienced during winter 22.38613 (g/m^3^), which also had the highest average concentration. Compared to the nitrate (NO_3_) flux, the lowest concentration of NH_4_–N was experienced in spring, while it was nitrate during summer.

**Figure 6:**
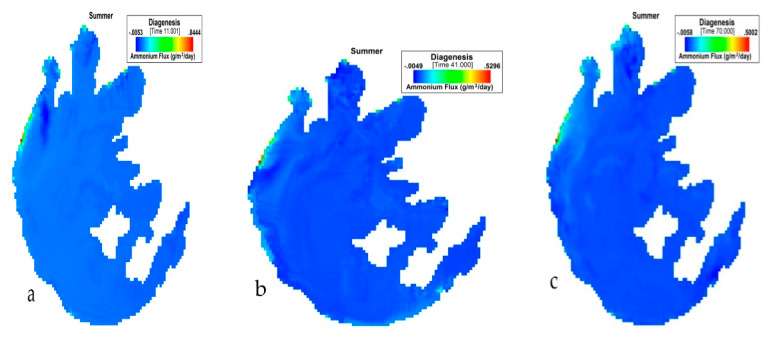
(**a**) 11th, (**b**) 41st, (**c**) 70th day simulation of NH_4_ flux variation during summer at Taihu.

Figure 6 presents the simulation of NH_4_ flux variation during summer in Taihu. During JJA, the NH_4_ flux variation had the lowest value of −0.0058 (g/m^2^/day) and an average of 0.053433 (g/m^2^/day). The highest value for this period was 0.844 (g/m^2^/day).

**Figure 7:**
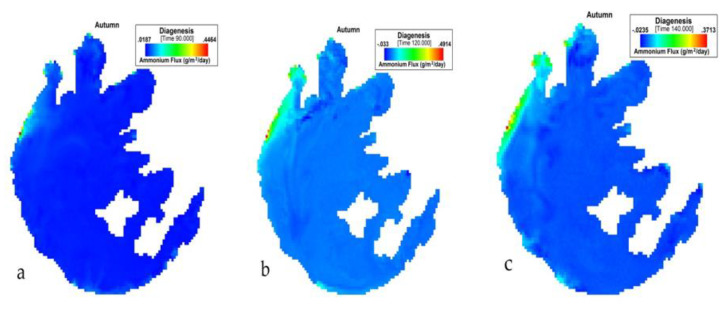
(**a**) 90th, (**b**) 120th, (**c**) 140th day simulation of NH_4_ flux variation during autumn at Taihu.

Figure 7 presents the simulation of NH_4_ flux variation during autumn in Taihu. During SON, the NH_4_ flux variation also had the lowest value of −0.0372 (g/m^2^/day) and an average of 0.02683 (g/m^2^/day). The highest value for this period was 0.03385 (g/m^2^/day).

**Figure 8:**
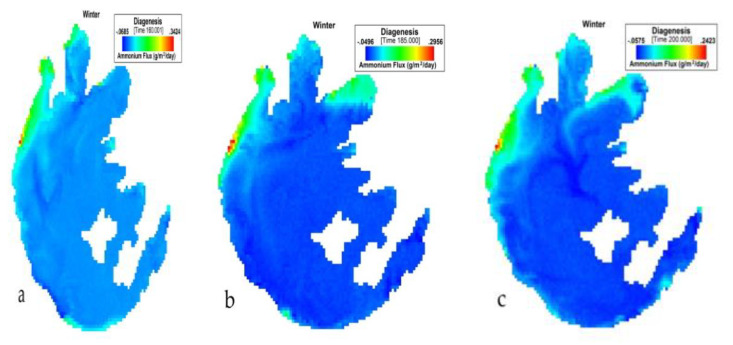
(**a**) 160th, (**b**) 185th, (**c**) 200th day simulation of NH_4_ flux variation during winter at Taihu.

Figure 8 presents the simulation of NH_4_ flux variation during winter in Taihu. During DJF, the NH_4_ flux variation had the lowest value of −0.0685 (g/m^2^/day) and an average of −0.01314 (g/m^2^/day). The highest value for this period was 0.3424 (g/m^2^/day).

**Figure 9:**
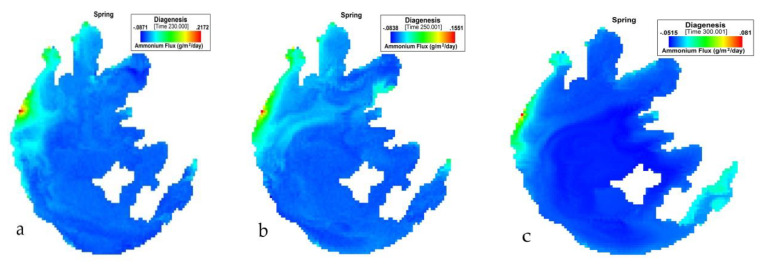
(**a**) 230th, (**b**) 250th, (**c**) 300th day simulation of NH_4_ flux variation during spring at Taihu.

Figure 9 presents the simulation of NH_4_ flux variation during spring in Taihu. During MAM, the NH_4_ flux variation had the lowest value of −0.0871 (g/m^2^/day) and an average of −0.046476667 (g/m^2^/day). The highest value for this period was 0.2172 (g/m^2^/day).

### 3.3. Calibration of the Lake Taihu Sediment Diagenesis Model with the Water Quality Model

The measured data averaged from 1.082 to 3.593 (mg/l), while the model data averaged from 0.907 to 3.158(mg/l) at Taihu. The relative error ranged from 24.377% to 49.417%.

## 4. Discussion

**Figure 10:**
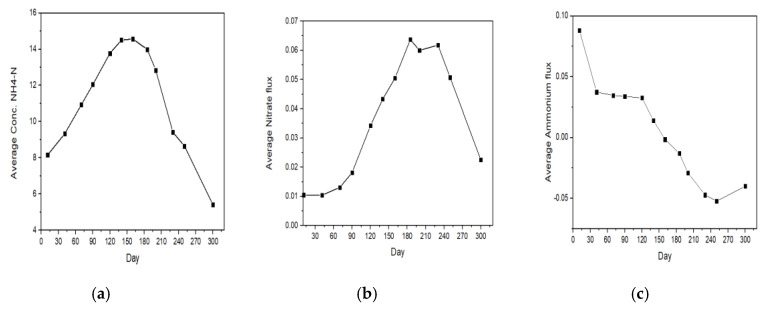
Seasonal variation of (**a**) average Conc NH_4_–N, (**b**) average nitrate (NO_3_); and (**c**) average ammonium (NH_4_) fluxes at Taihu, China.

The implications of the summary of results in Figure 10 explains that in summer, ammonium (NH_4_) fluxed out of the sediment with very little oxidation to nitrate (NO_3_). As a result, ammonium (NH_4_) accumulated in the water column. This is consistent with anoxic conditions. The flux of nitrate at that time was not negative (assuming that the positive flux means out of the sediment). Nitrate (NO_3_) was expected to flux into the sediment under anoxic conditions. Perhaps the concentration of nitrate (NO_3_) was very low in the water column at that time.

In autumn and winter, the sediment is most likely progressively oxidized. As a result, more of the sediment ammonium oxidized to nitrate. The flux of ammonium decreased, while the flux of nitrate (NO_3_) increased. The concentration of ammonium continued to increase until around day 150 because the flux of ammonium was still positive.

After day 150, the sediment became a sink for ammonium, implying that the oxidation of ammonium occurred in the sediment much faster than in the water column. The negative flux caused the ammonium concentrations to decrease. The continued oxidation of ammonium, however, continued to produce nitrate (NO_3_). The results were consistent. This would allow algal blooms to continue to thrive in the lake, creating a threat to water quality sustainability.

With the onset of low-oxygen conditions in spring, denitrification intensified, which consumed nitrate (NO_3_) by converting it predominantly to nitrogen gas. Part of it may be converted to ammonium via dissimilatory nitrate reduction to ammonium (DNRA).

Due to similar conditions of eutrophic Lake Taihu, Lake Okeechobee, and Chesapeake Bay, the pattern of this model was compared with Conc. NH_4_–N of Lake Okeechobee and the nitrate flux of Chesapeake Bay. From Lake Okeechobee, in July 1999, there was an increase in Conc. NH_4_–N in the sediment–water column from 0.03 in the water column to 2 (g/m^3^) in the sediment pore water. The lake’s pore water concentration NH_4_–N also continued to increase with depths below the sediment–water interface [18]. Additionally, at Chesapeake Bay, from the measured data, one half of the nitrate formed by nitrification of diagenetically produced ammonia escaped as nitrate flux to the overlying water.

Additionally, almost all nitrate produced by sediment nitrification denitrified to nitrogen gas [9]. There was an alignment of the pattern of the results and affirmed similar behavior and observations. The range of values of this model fell within the nitrate (NO_3_) flux of Chesapeake Bay 0.05–0.10 (g/m^2^/day) [9].

Furthermore, Elorn and Aulne, two small eutrophic estuaries in France, explained a spatio-temporal variability of the sediment organic matter recycling distribution of nitrate (NO_x_) and NH_4_^+^. The two estuaries experienced a large suboxic and anoxic mineralization at the sediments, causing rapid removal of very high bottom water concentrations of nitrate (NO_x_) and the large NH_4_^+^ increase at depth at all stations. Nitrate (NO_x_) concentrations decreased between winter and spring [36]. Hence, denitrification also occurred in these estuaries during spring.

On the archipelago of the Gulf of Finland, Baltic Sea resuspension promoted nitrification, with an indication seen in increased NOx fluxes at both stations at 7 m and 20 m (by 30% and 27% respectively) and a lowered NH_4_ flux (by 48%) at the 7 m station for the month of May and August [37]. In freshwater systems and other coastal bodies, NH_4_^+^ is recognized by most algal species, particularly Cyanophyta, as the preferred nitrogen source for growth [38,39]. While bloom severity within each subdivision on western Lake Erie is temporally and spatially unique, it increased over the study period of July to October 2002–2016 [40].

During autumn in Xiangxi Bay of the Three Gorges Reservoir, China, there is increasing nutrient loading from anthropogenic sources, which adequately decreases the ecological diversity [38].

However, seasonal bloom distribution for the period of 2002–2011 on Lake Winnipeg was consistent with light limitation in the south basin and lake circulation transporting bloom material toward the north-east shore, which could be the result of low oxygen at the sediment. Inter-annual variability in average bloom severity was related to both total phosphorus (TP) loadings and summer lake surface temperatures [29].

**Figure 11:**
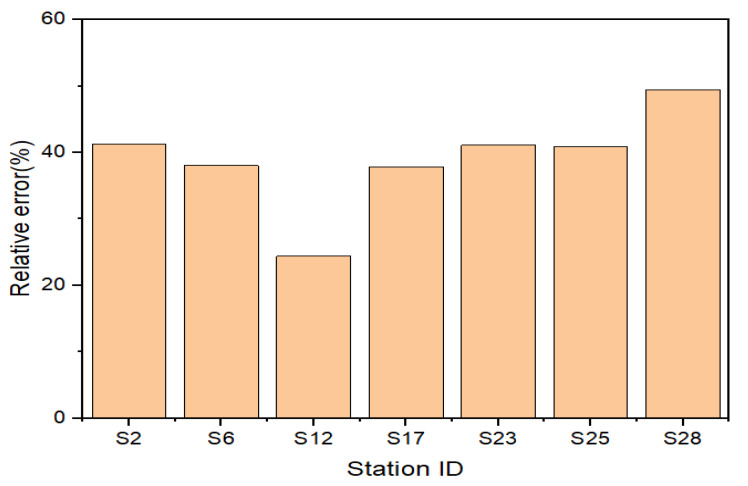
Average relative error for the water quality parameter total nitrogen (TN) at Taihu, China.

The evaluation results on Lake Chaohu revealed that effective HABs control in the lake depended on reducing P loading. Both internal and external P loading contributed significantly to HABs in the lake. August, May, and July were the hot moments for external P reduction. The Nanfei and Hangbu Rivers were hot spots for external P reduction [33]. Algae carbon is said to greatly consume oxygen at the benthic region, preventing denitrification from occurring. While nitrate denitrifies during spring and summer into nitrogen gas, phosphorus is not removed by this biochemical reaction. Future work would explore these areas. To ensure accuracy and quality of the simulated values, the results were calibrated. The total impression of the calibration (Figure 11) is that the model fluxes were in reasonable agreement [23,24]. The model was also found to be suitable because the average relative error for the TN was 38.137%.

## 5. Conclusions

Sediment flux has a significant contribution to lake management since it is established that it becomes a source for resuspension. During summer, the accumulation of ammonium (NH_4_) in the water column due to oxidation of nitrate (NO_3_) creates an environment for algal bloom. In autumn and winter, the elevated nitrate (NO_3_) and the concentration of ammonium levels increased the rate of algae producing toxins in the lake, which would cause immense contamination. As a result, an unhealthy aquatic environment is created for the organisms and reduces the suitability of water for human consumption.

Coupled with TN water quality measured data calibration at the lake, the relative error was below forty percent (40%), which makes the model suitable. The seasonal variation of sediment flux at Taihu provides the essence that creates awareness for more wholesome models on lakes for the sustainability of its water quality. Future studies should aim to simulate phosphate flux and provide measurements of the fluxes on the lake.

## Figures and Tables

**Table 1:** Environmental Fluid Dynamics Code (EFDC) sediment diagenesis model related variables and fluxes.

No	Parameter	Meaning	Unit	Range
**1**	Dp	Particle mixing apparent diffusion coefficient	m^2^/d	<0.001–0.5
**2**	θ_Dp_ThDp	Constant for temperature adjustment for Dp	-	1.07–1.117
**3**	Hsed	Diagenesis sediment thickness	m	0.10–0.20
**4**	W2	Sediment burial rate	cm/yr	0.02–1.0
**5**	θ_NH4_/ThNH_4_	Temperature coefficient for nitrification	-	1.076–1.127
**6**	K_N03,1_	Reaction velocity for denitrification in the aerobic layer	m/d	0.05–0.10
**7**	θ_NO3_/ThNO_3_	Temperature coefficient for denitrification	-	1.056–1.20
**8**	KPON1	Decay rate of PON at 20 °C for G1 class	1/d	0.019–0.066
**9**	ThKN1	Constant for temperature adjustment for KPON1	-	1.052–1.166
**10**	ThKN2	Constant for temperature adjustment for KPON2	-	1.052–1.166
**11**	θ_KM,NH4_	Temperature coefficient for nitrification half-saturation constant	-	1.125
**12**	KPON2	Decay rate of PON at 20degC for G2 class	1/d	0.0012–0.0088

**Table 2:** Shows the seasonal, ranges, and average concentration of NH_4_–N, sediment fluxes of NO_3_, and NH_4_ at Lake Taihu.

Time (Days)	Range (g/m^2^/Day)	Range (g/m^3^)	Range (g/m^2^/Day)	Average (g/m^2^/Day)	Range (g/m^3^)	Average (g/m^2^/Day)
	NO_3_ Flux	Conc. NH_4_-N	NH_4_ Flux	NO_3_ Flux	Conc.NH_4_-N	NH_4_ Flux
11th	−0.0162–0.01168	7.469671–18.81496	−0.005–0.844	0.0105	8.158883	0.08814
41st	−0.06622–0.01303	5.262655–19.0483	−0.0049–0.5296	0.0104	9.332679	0.03746
70th	−0.03165–0.01756	9.191248–17.47765	−0.0058–0.5002	0.0131	10.93654	0.0347
90th	−0.01081–0.0211	9.594671–19.50419	0.0187–0.4464	0.018149	12.0526	0.03385
120th	−0.04543–0.04031	4.2638–22.48205	−0.033–0.4914	0.0342	13.78247	0.03268
140th	−0.03398–0.04587	6.443372–22.31578	−0.0372–0.0746	0.0433	14.50533	0.01396
160th	−0.05547–0.5373	4.224813–22.38613	−0.0685–0.3424	0.0505	14.57058	−0.00146
185th	−0.06037–0.07113	4.278185–19.12033	−0.0496–0.2956	0.0637	13.99708	−0.01295
200th	−0.03079–0.0684	5.261141–18.6685	−0.0575–0.2429	0.0600	12.8486274	−0.02501
230th	−0.0163–0.07304	3.025818–13.67934	−0.0871–0.2172	0.0618	9.413582	−0.04715
250th	−0.0293–0.0649	5.151477–11.53736	−0.0838–0.1551	0.0507	8.65485	−0.05229
300th	−0.0102–0.03402	2.744903–9.846215	−0.0515–0.081	0.0223	5.405481	−0.03999
